# Efficacy and Safety of the Anti-mucosal Addressin Cell Adhesion Molecule-1 Antibody Ontamalimab in Patients with Moderate-to-Severe Ulcerative Colitis or Crohn’s Disease

**DOI:** 10.1093/ecco-jcc/jjad199

**Published:** 2023-12-14

**Authors:** Séverine Vermeire, Silvio Danese, William J Sandborn, Stefan Schreiber, Stephen Hanauer, Geert D’Haens, Peter Nagy, Manoj Thakur, Caleb Bliss, Fabio Cataldi, Martina Goetsch, Kenneth J Gorelick, Walter Reinisch

**Affiliations:** Department of Gastroenterology & Hepatology, University Hospitals Leuven, Leuven, Belgium; Inflammatory Bowel Diseases Center, Department of Gastroenterology, Humanitas Clinical and Research Center–IRCSS, and Department of Biomedical Sciences, Humanitas University, Milan, Italy; Division of Gastroenterology, University of California San Diego, La Jolla, CA, USA; Department of General Internal Medicine, Christian-Albrechts-Universität, Kiel, Germany; Department of Medicine [Gastroenterology and Hepatology], Northwestern University Feinberg School of Medicine, Chicago, IL, USA; Department of Gastroenterology and Hepatology, Amsterdam UMC, University of Amsterdam, Amsterdam, The Netherlands; Shire, a Takeda company, Zug, Switzerland; Shire, a Takeda company, Lexington, MA, USA; Takeda Pharmaceuticals, Lexington, MA, USA; Shire, a Takeda company, Lexington, MA, USA; Apellis Pharmaceuticals, Waltham, MA, USA; Shire, a Takeda company, Lexington, MA, USA; Landos Biopharma, Blacksburg, VA, USA; Shire, a Takeda company, Zug, Switzerland; Arena Pharmaceuticals, wholly owned subsidiary of Pfizer, Zurich, Switzerland; Zymo Consulting Group, Newtown Square, PA, USA; Department of Internal Medicine III, Division of Gastroenterology and Hepatology, Medical University of Vienna, Vienna, Austria

**Keywords:** Biomarkers, clinical trials, endoscopy

## Abstract

**Background and Aims:**

Ontamalimab is a fully human immunoglobulin G2 monoclonal antibody against mucosal addressin cell adhesion molecule-1, developed as treatment for inflammatory bowel disease.

**Methods:**

Six phase 3, multicentre, randomised, double-blind, placebo-controlled clinical trials compared efficacy and safety of ontamalimab [25 mg and 75 mg once every 4 weeks] with placebo in patients with moderate-to-severe ulcerative colitis or Crohn’s disease [two induction studies and one re-randomised maintenance study per condition]. This clinical trial programme was discontinued in 2020 for reasons unrelated to drug safety/efficacy; Crohn’s disease studies are described in the Supplementary data.

**Results:**

The induction [12-week] and maintenance [52-week] studies included 659 and 366 randomised patients, respectively. More patients who received ontamalimab induction than placebo achieved the primary endpoint of clinical remission at Week 12 [25 mg, 18.5% vs 15.8%, *p *= 0.617, 27.0% vs 12.5%, *p *= 0.027; 75 mg, 29.8% vs 15.8%, *p *= 0.018, 29.5% vs 12.5% *p *= 0.014]; significantly more patients who received ontamalimab maintenance therapy than placebo achieved Week 52 clinical remission [25 mg, 53.5% vs 8.2%, *p *<0.001; 75 mg, 40.2% vs 12.8%, *p *<0.001]. Endoscopic improvement was generally significantly different vs placebo [induction: 25 mg, 27.8% vs 21.1%, *p *= 0.253, 35.1% vs 12.5%, *p *= 0.001; 75 mg, 41.1% vs 21.1%, *p *= 0.002, 33.9% vs 12.5%, *p *= 0.003; maintenance: 25 mg, 56.3% vs 9.6%, *p *<0.001; 75 mg, 48.8% vs 15.1%, *p *<0.001]. Adverse event rates were similar between ontamalimab and placebo groups.

**Conclusions:**

Ontamalimab 75 mg was effective, with no safety concerns, as induction and maintenance therapy for patients with moderate-to-severe ulcerative colitis. [NCT03259334; NCT03259308; NCT03290781; NCT03559517; NCT03566823; NCT03627091]

## 1. Introduction

Inflammatory bowel disease [IBD] is a chronic inflammatory disorder of the gastrointestinal tract, of which ulcerative colitis [UC] and Crohn’s disease [CD] are the leading phenotypes.

Treatment of UC or CD aims to achieve and maintain remission, including alleviating symptoms and inducing endoscopic healing.^1^ Immunosuppressive therapies such as glucocorticoids and immunomodulators are commonly used to manage IBD^2,3^; however, these therapies are often associated with lack of response or intolerance. Moreover, the risks associated with long-term glucocorticoids use outweigh their benefits.^4^ Biologic therapies such as anti-tumour necrosis factor [anti-TNF] agents [infliximab, adalimumab, golimumab], anti-interleukin 12/23 agents [ustekinumab, risankizumab], and vedolizumab [anti-α4β7 integrin receptor] represent a more precise approach to promote mucosal healing.^4–6^ New small-molecule drug classes, such as Janus kinase inhibitors and sphingosine-1-phosphate receptor modulators, also provide therapeutic benefits for patients with IBD.^4,7^ Despite the availability of these treatment options, many patients have an inadequate response, lose response, or are unable to tolerate their treatment.^1,4^ Phase 3 studies of biologics in UC show induction remission rates of 17% to 27% and 1-year remission rates of under 50%, indicating a significant unmet medical need.^4,8^

Lymphocyte homing to the mucosal addressin cell adhesion molecule‐1 [MAdCAM-1] receptor represents a novel therapeutic target in UC and CD.^9–12^ Expression of MAdCAM‐1 is increased in the intestinal mucosal endothelium [in the high endothelial venules] under the conditions of chronic gastrointestinal inflammation found in patients with IBD, which facilitates lymphocyte infiltration of the gastrointestinal tract.^13–16^ The anti-inflammatory human immunoglobulin G2 monoclonal antibody ontamalimab inhibits binding of α4β7+ lymphocytes to MAdCAM-1 receptor-expressing sites by selectively binding with high affinity to MAdCAM-1.^17^ This differs from the α4β7-binding monoclonal antibody vedolizumab, approved for the treatment of UC and CD, and the α4β1-binding monoclonal antibody natalizumab, approved for the treatment of CD, which target integrins on circulating lymphocytes.^18,19^ By not binding directly to the α4β1 integrin, ontamalimab does not affect the activity of lymphocytes in the healthy central nervous system while still inhibiting the migration of lymphocytes into the gut, which potentially minimises off-target effects.^20^ In phase 2 studies, ontamalimab was reported to be well tolerated for up to 144 weeks in patients with moderate-to-severe UC, with good safety and efficacy [induction of remission].^21,22^ In patients with moderate-to-severe CD, ontamalimab was well tolerated, but clinical endpoint differences did not reach statistical significance compared with placebo.^23,24^

Here, we present the results from three phase 3 randomised controlled studies evaluating the efficacy and safety of ontamalimab for the induction and maintenance of remission in patients with moderate-to-severe UC. Results from a further three randomised controlled studies evaluating the efficacy and safety of ontamalimab for the induction and maintenance of remission in patients with moderate-to-severe CD are described in the Supplementary data.

## 2. Materials and Methods

### 2.1. Study design

Three phase 3 randomised, double-blind, placebo-controlled, parallel-group studies investigated the efficacy and safety of ontamalimab in patients with moderate-to-severe UC [induction studies NCT03259334 and NCT03259308; maintenance study NCT03290781]. Separately, three phase 3 randomised, double-blind, placebo-controlled, parallel-group studies investigated the efficacy and safety of ontamalimab in patients with moderate-to-severe CD [induction studies NCT03559517 and NCT03566823; maintenance study NCT03627091].

On May 29, 2020, Takeda announced the decision to discontinue the ontamalimab clinical trial programme in UC and CD for reasons unrelated to the safety or efficacy of the drug.^25^ This primarily affected recruitment for the CD trials, and so CD trial methodology and results are provided in the Supplementary data.

**Figure 1:**
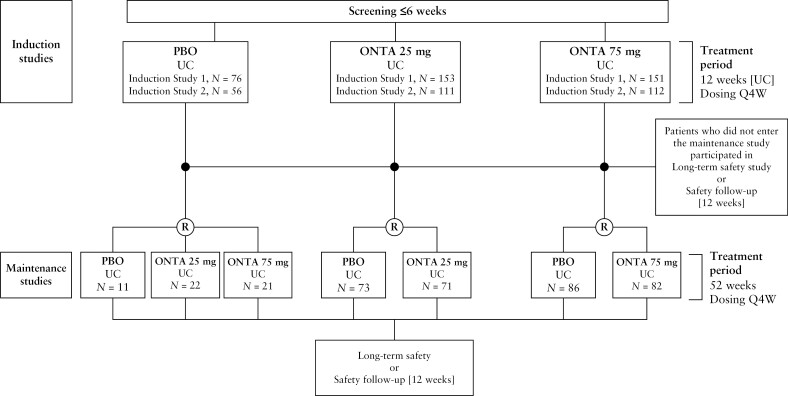
Study flow. Patients with UC received ontamalimab or placebo once every 4 weeks [randomised set]. Two patients in the Induction Study 1 25 mg treatment group were randomised but never received treatment and were excluded from the full analysis set. ONTA, ontamalimab; PBO, placebo; Q4W, every 4 weeks; R, randomisation; UC, ulcerative colitis.

The UC studies were conducted between December 2017 and September 2021 across 404 sites, in North America, Europe, the Middle East and Africa, Latin America, and Asia-Pacific [patients from Japan were included in one induction study] [Supplementary Table 1]. Two similarly designed induction studies were performed in patients with UC [Induction Study 1 [NCT03259334] and Induction Study 2 [NCT03259308]]. A summary of patient flow in these studies is given in Figure 1.

Patients in the induction studies were at least 16 years of age but younger than 80 years at the time of informed consent/assent; had a documented diagnosis of moderate-to-severe UC at least 3 months before screening; and had an inadequate response to, a lost response to, or an intolerance to at least one conventional treatment, such as mesalamine, an immunosuppressant, or an anti-TNF agent. To participate in the maintenance studies, patients had to have completed treatment in the induction studies and had to have achieved a clinical response [definitions are provided in Supplementary Methodology]. Further inclusion and exclusion criteria are provided in Supplementary Table 2.

Patients participating in the induction studies entered a screening period of up to 6 weeks, after which eligible patients could enter a 12-week treatment period. During the treatment period, patients were randomised 2:2:1 to receive ontamalimab 25 mg, ontamalimab 75 mg, or placebo, via subcutaneous [SC] injections, once every 4 weeks. Patients who achieved clinical response at the end of induction could enter the maintenance study. Patients who did not show a clinical response at the end of the induction treatment period could participate in a long-term safety [LTS] extension study [NCT03283085; trial is ongoing]. Patients who withdrew early from the treatment period or who did not wish to enter the maintenance or LTS extension study continued into a 16-week safety follow-up period.

For patients who entered the maintenance study [treatment for up to 52 weeks], Week-12 visits from the induction studies were used as the maintenance study baseline. During the treatment period, patients who had previously received ontamalimab during the induction study were randomised 1:1 to receive ontamalimab [at their previous dose] or placebo. Patients who had received placebo in the induction study were randomised 2:2:1 in the maintenance study to receive ontamalimab 25 mg, ontamalimab 75 mg, or placebo [these patients did not contribute to primary efficacy analyses but contributed to safety analyses]. Patients who completed the maintenance study could enter the LTS extension study. Patients who experienced disease worsening/treatment failure [defined by protocol as sustained symptomatic worsening, confirmed by endoscopy] were able to exit the study and enter the LTS to receive active treatment. Patients who discontinued early or did not enter the LTS extension study entered a 16-week safety follow-up period [reduced to 12 weeks in the LTS extension study].

In the induction studies, patients were stratified based on prior anti-TNF treatment status [naive or experienced] and glucocorticoid use at baseline [receiving glucocorticoids or not at baseline]. In the maintenance study, stratification was additionally based on the degree of clinical response in the induction study [remission achieved or not].

### 2.2. Assessments and outcomes

The primary efficacy endpoint in the induction and maintenance studies was clinical remission at Week 12 [induction] or Week 52 [maintenance]. Clinical remission was defined as a composite score of stool frequency [SF] subscore of 0 or 1 with at least a 1-point change from baseline, rectal bleeding [RB] subscore of 0, and endoscopic subscore of 0 or 1 reported by patients using daily e-diary and centrally read endoscopy [ie, the adapted Mayo score]. Key secondary endpoints were endoscopic improvement, symptomatic remission, clinical response, and mucosal healing [defined as a centrally read endoscopic subscore of 0 or 1 and a centrally read Geboes score^26^ of ≤2] at Week 12 or Week 52. Definitions for all secondary endpoints are given in Supplementary Table 3. Biomarker and pharmacodynamic endpoints assessed were changes from baseline in faecal calprotectin, serum C-reactive protein [CRP], and serum soluble MAdCAM. In the maintenance study, key secondary endpoints included sustained remission [remission at the Week 52 visit among patients who were in remission at the time of baseline in the maintenance study], and glucocorticoid-free symptomatic remission and glucocorticoid-free clinical remission among patients receiving glucocorticoids at baseline [symptomatic/clinical remission at Week 52 in addition to not requiring any treatment with glucocorticoids for at least 4 weeks prior to the Week 52 visit]. The glucocorticoid tapering regimen is defined in Supplementary Methodology.

### 2.3. Safety endpoints

Safety endpoints included adverse events [AEs], changes in clinical laboratory test results, and the presence of anti-drug antibodies [ADAs] and neutralising antibodies [NAbs]. Serum samples were also collected for John Cunningham virus antibody testing if patients showed symptoms suggestive of progressive multifocal leukoencephalopathy [PML] following targeted neurological assessments.

### 2.4. Statistical analysis

Efficacy data were reported using the full analysis set, defined as all patients in the randomised set who had received at least one dose of investigational product in the induction studies, and all patients in the randomised set who had received at least one dose of investigational product in the maintenance study and who were previously treated with ontamalimab in the induction studies. Safety data were reported using the safety set [all patients who had received at least one dose of ontamalimab]. Power calculations for each trial were performed and included in Supplementary Methodology.

The primary and key secondary endpoints were compared for each active treatment group with the placebo group, using a Cochran–Mantel–Haenszel χ^2^ test stratified by actual status of prior anti-TNF treatment and glucocorticoid use at baseline [all studies] and remission induction [maintenance studies]. Patients with missing data at Week 12 or Week 52, or with intercurrent events before these time points, were considered failures [ie, composite estimand approach^27^]. Statistical significance was determined using the hierarchical approach utilised to propagate alpha from the primary to key secondary endpoints, and between the two ontamalimab treatment groups and placebo comparisons. Details of multiplicity control are provided in Supplementary Methodology. Post hoc analyses of remission based on composite score by patients’ prior anti-TNF treatment status [naïve or experienced] were performed. Descriptive statistics (mean and standard deviation [SD]) were used to summarise the change from baseline in biomarkers.

AEs were classified into preferred terms, and summarised into frequency and type of AE using Version 19.1 of Medical Dictionary for Regulatory Activities [MedDRA]. Descriptive statistics were used to summarise immunogenicity data.

All studies were conducted in accordance with International Council for Harmonisation [ICH] Good Clinical Practice [GCP] Guideline E6 [1996] and E6 R2 [2017], EU Directive 2001/20/EC, the principles of the Declaration of Helsinki, as well as other applicable national ethical and legal requirements, and all patients provided written informed consent or assent. The study protocols, protocol amendments, final approved informed consent/assent documents, relevant supporting information, and all types of subject recruitment information were submitted by the investigator to the Institutional Review Board [IRB] or Independent Ethics Committee [IEC] and approved by the IRB/IEC and regulatory agency [as appropriate] prior to initiation of studies.

## 3. Results

### 3.1. Patient disposition, baseline demographics, and characteristics

Overall, 659 and 366 randomised patients with UC were included in the induction [Induction Study 1, *n* = 380; Induction Study 2, *n* = 279] and maintenance studies, respectively [Figure 1].

Patients were 16–78 years of age [Induction Study 1, mean 38.4 years, range 16–78 years; Induction Study 2, mean 43.3 years, range 18–77 years; maintenance study, mean 40.9 years, range 16–78 years]. Baseline demographics and characteristics of patients are summarised in Table 1. The premature discontinuation of the studies meant the intended number of patients was not recruited for any of the three studies; 38–53% of the intended number of patients were recruited.

**Table 1:** Baseline demographics and characteristics for patients with ulcerative colitis [full analysis set].

	Induction Study 1			Induction Study 2			Maintenance study			
	Placebo *N* = 76	Ontamalimab 25 mg *N* = 151	Ontamalimab 75 mg *N* = 151	Placebo *N* = 56	Ontamalimab 25 mg *N = *111	Ontamalimab 75 mg *N* = 112	Ontamalimab 25 mg/placebo *N* = 73	Ontamalimab 25 mg/ontamalimab 25 mg *N* = 71	Ontamalimab 75 mg/placebo *N* = 86	Ontamalimab 75 mg/ ontamalimab 75 mg *N* = 82
Age [years]										
Mean [SD]	38.3 [13.33]	39.4 [13.90]	41.2 [14.75]	41.6 [13.50]	43.5 [14.16]	43.9 [13.08]	43.3 [15.40]	41.2 [13.17]	42.0 [13.81]	42.3 [14.21]
Min, max	16, 73	16, 78	16, 75	19, 74	18, 77^a^	20, 71	19, 78	16, 73	17, 72	20, 71
Sex, *n* [%]										
Male	43 [56.6]	95 [62.9]	89 [58.9]	33 [58.9]	65 [58.6]	67 [59.8]	46 [63.0]	42 [59.2]	51 [59.3]	47 [57.3]
Race, *n* [%]										
Asian	1 [1.3]	10 [6.6]	9 [6.0]	11 [19.6]	10 [9.0]	14 [12.5]	5 [6.8]	4 [5.6]	5 [5.8]	8 [9.8]
Black or African American	1 [1.3]	2 [1.3]	5 [3.3]	2 [3.6]	4 [3.6]	1 [0.9]	3 [4.1]	1 [1.4]	2 [2.3]	2 [2.4]
White	72 [94.7]	134 [88.7]	136 [90.1]	41 [73.2]	85 [76.6]	88 [78.6]	58 [79.5]	62 [87.3]	77 [89.5]	69 [84.1]
Other	2 [2.6]	5 [3.3]	1 [0.7]	2 [3.6]	12 [10.8]	9 [8.0]	7 [9.6]	4 [5.6]	2 [2.3]	3 [3.7]
Disease duration, years^b^										
Mean [SD]	7.2 [6.5]	6.7 [7.2]	7.1 [7.4]	6.9 [8.0]	6.6 [6.9]	7.2 [8.0]	7.7 [7.2]	6.9 [7.2]	6.6 [6.5]	8.6 [9.6]
Total Mayo severity										
* n*	76	149	150	55	109	112	72	70	86	81
Mean [SD]	9.0 [1.25]	9.0 [1.29]	9.0 [1.33]	9.0 [1.66]	9.1 [1.51]	9.3 [1.41]	8.9 [1.37]	9.0 [1.31]	9.0 [1.31]	8.9 [1.41]
Endoscopy findings, *n* [%]										
0	0	0	0	0	0	0	0	0	0	0
1	0	0	0	0	0	0	0	0	0	0
2	26 [34.2]	55 [36.4]	62 [41.1]	23 [41.1]	41 [36.9]	38 [33.9]	30 [41.1]	33 [46.5]	33 [38.4]	38 [46.3]
3	50 [65.8]	96 [63.6]	89 [58.9]	33 [58.9]	69 [62.2]	74 [66.1]	43 [58.9]	37 [52.1]	53 [61.6]	44 [53.7]
Missing	-	-	-	0	1 [0.9]	0	0	1 [1.4]	0	0
Endoscopy findings at maintenance baseline, *n* [%]										
0	-	-	-	-	-	-	13 [17.8]	7 [9.9]	17 [19.3]	19 [23.2]
1	-	-	-	-	-	-	25 [34.2]	27 [38.0]	31 [36.0]	28 [34.1]
2	-	-	-	-	-	-	25 [34.2]	18 [25.4]	22 [25.6]	19 [23.2]
3	-	-	-	-	-	-	10 [13.7]	19 [26.8]	16 [18.6]	16 [19.5]
Baseline faecal calprotectin [mg/kg]										
* n*	68	136	125	49	90	97	63	64	69	70
Mean [SD]	3511.0 [4225.2]	3158.9 [3484.7]	3258.6 [4078.1]	3720.5 [5044.6]	3498.9 [5350.7]	3247.4 [3985.8]	2773.0 [2687.7]	3535.5 [4788.8]	2692.8 [2711.6]	3642.3 [5224.2]
Baseline CRP [mg/L]										
* n*	73	149	143	54	108	108	7 [/73]	5 [/69]	8 [/81]	2 [/75]
Mean [SD]	10.8 [27.1]	12.2 [32.3]	6.2 [12.8]	6.6 [13.0]	10.1 [26.6]	8.6 [12.3]	20.2 [44.9]	5.3 [5.0]	1.1 [0.5]	4.3 [4.0]
Status of anti-TNF experience, *n* [%]										
Experienced	22 [28.9]	47 [31.1]	44 [29.1]	12 [21.4]	24 [21.6]	24 [21.4]	17 [23.3]	19 [26.8]	18 [20.9]	14 [17.1]
Naive	54 [71.1]	104 [68.9]	107 [70.9]	44 [78.6]	87 [78.4]	88 [78.6]	56 [76.7]	52 [73.2]	68 [79.1]	68 [82.9]
Anti-TNF failure,^c^*n* [%]										
	15 [19.7]	27 [17.9]	33 [21.9]	9 [16.1]	19 [17.1]	18 [16.1]	10 [13.7]	11 [15.5]	14 [16.3]	7 [8.5]
Systemic or topical GC use at baseline, *n* [%]										
	33 [43.4]	64 [42.4]	63 [41.7]	20 [35.7]	41 [36.9]	42 [37.5]	27 [37.0]	22 [31.0]	32 [37.2]	30 [36.6]
Maximum prior treatment experience,^d^*n* [%]										
5-ASA experienced	4 [5.3]	21 [13.9]	17 [11.3]	9 [16.1]	25 [22.5]	24 [21.4]	14 [19.2]	15 [21.1]	17 [19.8]	12 [14.6]
GC experienced	23 [30.3]	50 [33.1]	63 [41.7]	22 [39.3]	35 [31.5]	42 [37.5]	27 [37.0]	15 [21.1]	31 [36.0]	42 [51.2]
Topical GC experienced	6 [7.9]	9 [6.0]	11 [7.3]	7 [12.5]	9 [8.1]	9 [8.0]	8 [11.0]	0 [0.0]	6 [7.0]	9 [11.0]
Systemic GC experienced	17 [22.4]	41 [27.2]	52 [34.4]	15 [26.8]	26 [23.4]	33 [29.5]	19 [26.0]	15 [21.1]	25 [29.1]	33 [40.2]
Immunosuppressant experienced or biologic failure	37 [48.7]	57 [37.7]	43 [28.5]	17 [30.4]	39 [35.1]	31 [27.7]	24 [32.9]	34 [47.9]	27 [31.4]	21 [25.6]
Immunosuppressant experienced and biologic failure	12 [15.8]	23 [15.2]	28 [18.5]	8 [14.3]	12 [10.8]	15 [13.4]	8 [11.0]	7 [9.9]	11 [12.8]	7 [8.5]

Baseline values were the last value collected before the first dose of study treatment.

^a^The age of one patient who was 17.84 years of age, enrolled in the ontamalimab 25 mg treatment group, was rounded up to 18 years for the purpose of establishing a minimum value.

^b^Disease duration was the number of years from the date of UC diagnosis to the date of informed consent.

^c^Anti-TNF failure included intolerance.

^d^Patients were counted once at the maximum prior treatment experienced, with the categories representing increasing prior treatment experienced.

5-ASA, aminosalicylate; anti-TNF, anti-tumour necrosis factor; CRP, C-reactive protein; GC, glucocorticoid; max, maximum; min, minimum; SD, standard deviation; UC, ulcerative colitis.

#### 3.1.1. Primary endpoint—induction studies [Week 12] and maintenance study [Week 52]

**Figure 2:**
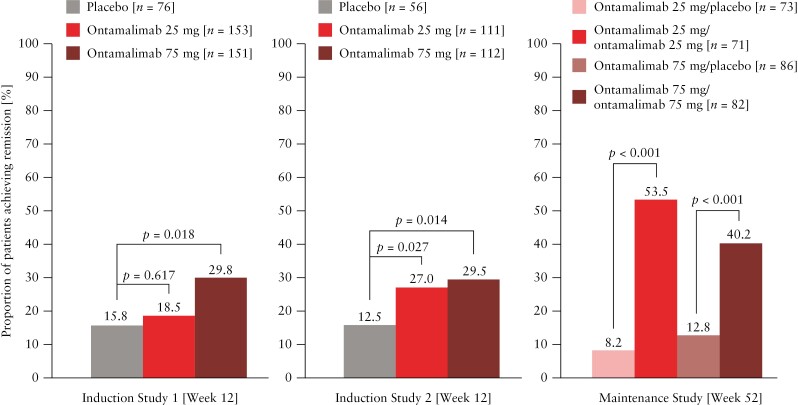
Proportion of patients with UC achieving clinical remission [randomised set]. Clinical remission was defined as a stool frequency subscore of 0 or 1 with at least a 1-point change from baseline and a rectal bleeding subscore of 0 and an endoscopic subscore of 0 or 1. Patients with missing data at Week 12 or with intercurrent events before Week 12 were considered failures. UC, ulcerative colitis.

In the induction studies, more patients who received ontamalimab 25 mg or 75 mg achieved clinical remission at Week 12 than patients who received placebo. This difference was statistically significant for patients receiving 75 mg in Induction Studies 1 and 2 (45 [29.8%] vs 12 [15.8%], *p *= 0.018; and 33 [29.5%] vs 7 [12.5%], *p *= 0.014, respectively) and for patients receiving 25 mg in Induction Study 2 (30 [27.0%] vs 7 [12.5%], *p *= 0.027) [Figure 2]. In the maintenance study, statistically significantly more patients who received ontamalimab 25 mg or 75 mg achieved clinical remission at Week 52 than patients who received ontamalimab/placebo (25 mg: 38 [53.5%] vs 6 [8.2%], *p *<0.001; 75 mg: 33 [40.2%] vs 11 [12.8%], *p *<0.001).

Regarding clinical remission by baseline anti-TNF status, in the induction studies, among anti-TNF experienced patients, six [12.8%] and seven [29.2%] in the ontamalimab 25 mg groups, six [13.6%] and seven [29.2%] in the ontamalimab 75 mg groups, and six [27.3%] and three [25.0%] in the placebo groups, were in remission at Week 12. Among anti-TNF naïve patients, 22 [21.2%] and 23 [26.4%] in the ontamalimab 25 mg groups, 39 [36.4%] and 26 [29.5%] patients in the ontamalimab 75 mg groups, and six [11.1%] and four [9.1%] in the placebo groups, were in remission at Week 12. At maintenance study baseline, 148 [75.5%] patients who received ontamalimab and 132 [77.6%] patients who received placebo were anti-TNF naïve. Post hoc analyses showed results similar to the full data analyses in the two subpopulations of anti-TNF experienced and anti-TNF naïve patients. Significantly more anti-TNF experienced patients were in remission at Week 52 in the 25 mg ontamalimab (10 [52.6%] patients) and 75 mg ontamalimab (4 [28.6%] patients) groups compared with placebo (1 [2.9%] patient; *p* <0.001 and *p* = 0.006, respectively). Significantly more anti-TNF naïve patients were in remission at Week 52 in the 25 mg ontamalimab (28 [53.8%] patients) and 75 mg ontamalimab (29 [42.6%] patients) treatment groups compared with placebo (16 [12.9%]; both *p* <0.001). Interestingly, among placebo-treated patients, more anti-TNF naïve [12.9%] than anti-TNF experienced [2.9%] patients were in remission at Week 52.

#### 3.1.2. Secondary endpoints—induction studies [Week 12]

In the induction studies, more patients who received ontamalimab achieved endoscopic improvement than patients who received placebo [Table 2]. This difference was statistically significant for patients receiving ontamalimab 75 mg in Induction Studies 1 and 2 [*p *= 0.002 and *p *= 0.003, respectively] and for patients receiving ontamalimab 25 mg in Induction Study 2 [*p *<0.001].

**Table 2:** Key secondary endpoints for patients with ulcerative colitis [full analysis set].

	Induction study 1			Induction study 2			Maintenance study			
	Placebo *N* = 76	Ontamalimab 25 mg *N* = 151	Ontamalimab 75 mg *N* = 151	Placebo *N* = 56	Ontamalimab 25 mg *N* = 111	Ontamalimab 75 mg *N* = 112	Ontamalimab 25 mg/ placebo *N* = 73	Ontamalimab 25 mg/ontamalimab 25 mg *N* = 71	Ontamalimab 75 mg/placebo *N* = 86	Ontamalimab 75 mg/ontamalimab 75 mg *N* = 82
Endoscopic improvement^a^ at Week 12 [induction studies] and Week 52 [maintenance study]^b^										
*n* [%]	16 [21.1]	42 [27.8]	62 [41.1]	7 [12.5]	39 [35.1]	38 [33.9]	7 [9.6]	40 [56.3]	13 [15.1]	40 [48.8]
*p-*value	-	0.253	0.002	-	0.001	0.003	-	<0.001	-	<0.001
Sustained remission										
*n* [%]	-	-	-	-	-	-	4 [5.5]	23 [32.4]	7 [8.1]	22 [26.8]
*p-*value	-	-	-	-	-	-	-	<0.001	-	<0.001
Clinical response at Week 12 [induction studies] and Week 52 [maintenance study]										
*n* [%]	34 [44.7]	76 [50.3]	99 [65.6]	16 [28.6]	67 [60.4]	64 [57.1]	15 [20.5]	49 [69.0]	20 [23.3]	47 [57.3]
*p-*value	-	0.440	0.002	-	<0.001	<0.001	-	<0.001	-	<0.001
Mucosal healing at Week 12 [induction studies] and Week 52 [maintenance study]										
n [%]	13 [17.1]	35 [23.2]	51 [33.8]	6 [10.7]	35 [31.5]	30 [26.8]	6 [8.2]	37 [52.1]	11 [12.8]	29 [35.4]
*p-*value	-	0.286	0.005	-	0.002	0.017	-	<0.001	-	<0.001
Glucocorticoid-free symptomatic remission^c^										
*n* [%]	-	-	-	-	-	-	1 [3.7]	12 [54.5]	3 [9.4]	12 [40.0]
*p*-value	-	-	-	-	-	-	-	<0.001	-	0.005
Glucocorticoid-free clinical remission^d^										
*n* [%]	-	-	-	-	-	-	0 [0.0]	8 [36.4]	2 [6.3]	10 [33.3]
*p-*value	-	-	-	-	-	-	-	<0.001		<0.005

^a^Endoscopic improvement was defined as centrally read endoscopic subscore 0 or 1 [modified, excludes friability].

^b^There is no overall placebo rate given in this study owing to the design and how the randomisation was performed; *p*-values refer to corresponding ontamalimab/placebo subgroups.

^c^Glucocorticoid-free symptomatic remission was defined as symptomatic remission at Week 52 in addition to not requiring any treatment with glucocorticoids for at least 4 weeks prior to the Week 52 visit. Results only include patients receiving glucocorticoids.

^d^Glucocorticoid-free clinical remission was defined as clinical remission at Week 52 in addition to not requiring any treatment with glucocorticoids for at least 4 weeks prior to the Week 52 visit. Results only include patients receiving glucocorticoids.

Symptomatic remission was achieved by more patients receiving ontamalimab 25 mg [40.4% and 45.0%] or 75 mg [50.3% and 50.0%] than patients who received placebo [38.2% and 17.9%] in the induction studies. This difference was statistically significant for patients receiving ontamalimab 75 mg [*p *<0.001] and 25 mg [*p *<0.001] in Induction Study 2.

Clinical response was achieved by more patients receiving ontamalimab 25 mg or 75 mg than patients who received placebo in the induction studies. This difference was statistically significant for patients receiving ontamalimab 75 mg in Induction Studies 1 and 2 [*p *= 0.002 and *p *<0.001, respectively] and for patients receiving ontamalimab 25 mg in Induction Study 2 [*p *<0.001].

More patients receiving ontamalimab 25 mg and 75 mg had mucosal healing than patients who received placebo [Table 2]. This difference was statistically significant for patients receiving ontamalimab 75 mg in Induction Studies 1 and 2 [*p *= 0.005 and *p *= 0.017, respectively] and patients receiving ontamalimab 25 mg in Induction Study 2 [*p *= 0.002].

Among patients who received ontamalimab 75 mg or 25 mg in Induction Study 2, reductions from baseline were observed in faecal calprotectin [*p* <0.001 for both doses] and serum CRP [*p* = 0.036 and *p* = 0.026, respectively]. Serum soluble MAdCAM was reduced from baseline with both doses compared with placebo in Induction Studies 1 and 2 [all *p* <0.01] [Supplementary Table 4]. In contrast, numerical reductions in faecal calprotectin and serum CRP and little change in serum soluble MAdCAM were observed in patients receiving placebo in both induction studies.

#### 3.1.3. Secondary endpoints—maintenance study [Week 52]

Both doses of ontamalimab significantly outperformed placebo in all primary and key secondary endpoints, including endoscopic improvement, sustained remission, sustained clinical response, mucosal healing, and glucocorticoid-free remission [Table 2]. Patients receiving ontamalimab had reductions in faecal calprotectin [greater reductions observed at 25 mg than at 75 mg], and patients receiving 75 mg had the greatest reductions in serum CRP [Supplementary Table 4].

#### 3.1.4. Safety

During the induction studies, 44.4% of patients receiving ontamalimab reported treatment-emergent AEs [TEAEs; Induction Study 1, 49.7%; Induction Study 2, 37.2%] as did 42.4% of patients receiving placebo [Induction Study 1, 47.4%; Induction Study 2, 35.7%] [Table 3]. Among patients receiving ontamalimab, the proportion reporting TEAEs was smaller with the 75 mg dose [Induction Study 1, 45.7%; Induction Study 2, 33.0%] than with the 25 mg dose [Induction Study 1, 53.6%; Induction Study 2, 41.4%]. During the maintenance study, TEAEs were reported by 58.7% of patients receiving ontamalimab and 63.5% of patients receiving placebo. Most TEAEs were mild or moderate in severity and did not lead to discontinuation. Of the TEAEs reported in the induction studies, 8.2% of those reported by patients receiving ontamalimab [Induction Study 1, 10.3%; Induction Study 2, 5.4%] were considered related to ontamalimab, as were 9.9% of the TEAEs reported by patients receiving placebo [Induction Study 1, 7.9%; Induction Study 2, 12.5%]. In the maintenance study, 12.8% of TEAEs recorded for patients receiving ontamalimab were considered related to ontamalimab, and 9.4% in patients receiving placebo. Only 26 patients (eight receiving placebo [6.1%]; 18 receiving ontamalimab [3.4%]) discontinued the induction studies, and 19 patients (12 receiving placebo [7.1%]; seven receiving ontamalimab [3.6%]) discontinued the maintenance study; all discontinuations were due to TEAEs. The most common reason for discontinuation in patients treated with either ontamalimab or placebo was UC [induction studies, 10 events in nine patients [1.7%] receiving ontamalimab, and six patients [4.5%] receiving placebo; maintenance study, 12 events in four patients [2.0%] receiving ontamalimab and eight patients [4.7%] receiving placebo). Injection site reactions [haematoma, pain, and urticaria] were reported by fewer than 1% of patients receiving ontamalimab or placebo.

**Table 3:** Adverse events reported for patients with ulcerative colitis [safety set].

	Induction study 1			Induction study 2			Maintenance study		
	Placebo *N* = 76	Ontamalimab 25 mg *N* = 151	Ontamalimab 75 mg *N* = 151	Placebo *N* = 56	Ontamalimab 25 mg *N* = 111	Ontamalimab 75 mg *N* = 112	Placebo *N* = 170	Ontamalimab 25 mg *N* = 93	Ontamalimab 75 mg *N* = 103
Any TEAE, *n* [%]	36 [47.4]	81 [53.6]	69 [45.7]	20 [35.7]	46 [41.4]	37 [33.0]	108 [63.5]	52 [55.9]	63 [61.2]
Serious TEAE, *n* [%]	5 [6.6]	10 [6.6]	8 [5.3]	4 [7.1]	5 [4.5]	3 [2.7]	16 [9.4]	9 [9.7]	4 [3.9]
TEAE related to study drug, *n* [%]	6 [7.9]	14 [9.3]	17 [11.3]	7 [12.5]	6 [5.4]	6 [5.4]	16 [9.4]	13 [14.0]	12 [11.7]
TEAE leading to study discontinuation, *n* [%]	3 [3.9]	8 [5.3]	5 [3.3]	5 [8.9]	4 [3.6]	1 [0.9]	12 [7.1]	6 [6.5]	1 [1.0]
TEAE leading to death, *n* [%]	0 [0]	0 [0]	1 [0.7]	0 [0]	0 [0]	0 [0]	2 [0.1]	0 [0]	0 [0]

TEAE, treatment-emergent adverse event.

No confirmed cases of PML were identified in the induction or maintenance studies. No hypersensitivity events were reported for patients in the induction studies. In the maintenance study, one potential treatment-emergent drug hypersensitivity event was reported for one patient receiving ontamalimab 25 mg [1.1%, *N* = 93]. There were no notable mean changes in haematology, chemistry, or urinalysis parameters over time in the induction or maintenance studies, or between patients receiving ontamalimab or placebo.

One patient died during the safety follow-up period of Induction Study 1: a 17-year-old, White, male patient with a 7-year history of UC in the ontamalimab 75 mg treatment group who was hospitalised and received a diagnosis of the serious AE of T-cell precursor lymphoblastic lymphoma 17 days after the patient’s last dose of ontamalimab at Week 4, and died 107 days later. Previous and concomitant treatments included azathioprine, mercaptopurine, and prednisone. The event was not considered by the investigator to be related to ontamalimab. Two patients [receiving placebo] died during the maintenance study, and the causes of death were myocardial infarction and sudden cardiac death. A 64-year-old, White, male patient with a history of UC, with concomitant medications of mesalamine, azathioprine, a glucocorticoid, and metformin, received ontamalimab 75 mg for 12 weeks in the induction study and was re-randomised in the maintenance study to receive placebo, and died [myocardial infarction]. Study drug exposure was 157 days. The sudden cardiac death case was a 60-year-old, White, female patient with a history of UC and chronic pancreatitis, who had received concomitant glucocorticoid treatment and had received ontamalimab 75 mg for 12 weeks in the induction study, and was re-randomised to receive placebo in the maintenance study. Study drug exposure was 137 days.

The proportions of patients who tested positive for ADAs between baseline and Week 12 of the induction studies were similar between patients who received ontamalimab and those who received placebo [Supplementary Table 5]. No patient receiving ontamalimab was positive for NAbs in the induction studies. During the maintenance study, 26 patients [13.3%] who received ontamalimab and 27 patients [15.9%] who received placebo developed ADAs or boosted their ADAs. None of the patients in the maintenance study who tested positive for ADAs tested positive for NAbs.

## 4. Discussion

Three clinical studies, including two induction studies and one maintenance study, have assessed the efficacy and safety of ontamalimab 25 mg or 75 mg compared with placebo separately in patients with moderate-to-severe UC. A further three studies were initiated or planned in patients with moderate-to-severe CD. However, owing to the early termination of the programme in UC and CD, the CD studies had included only a small number of patients and so the results of these are presented only in the Supplementary data.

In patients with UC, ontamalimab as induction or maintenance therapy was associated with greater efficacy than placebo across primary and secondary outcomes. More patients with UC who received ontamalimab 25 mg or 75 mg experienced clinical remission, endoscopic improvement, symptomatic remission, clinical response, and mucosal healing after 12 and 52 weeks of treatment than patients who received placebo. Although these differences were not significant for patients receiving ontamalimab 25 mg in one induction study, the reported changes are consistent with the results from previous phase 2 studies of ontamalimab.^21,24^ Glucocorticoid-free symptomatic and clinical remission at Week 52 with ontamalimab suggests that patients may respond to ontamalimab without reliance on glucocorticoids, reducing the health risks associated with glucocorticoid use. Clinical responses achieved during the induction period were sustained during treatment maintenance. The maintenance results are generally consistent with clinical trial outcomes with small molecules and biologics in patients with UC, for which improved rates of clinical remission, mucosal healing, or glucocorticoid-free remission have been reported compared with placebo.^5–7,11,21^ A more consistent pattern of efficacy was also demonstrated in the present studies than previously reported for the gut-targeted anti-β7 integrin monoclonal antibody etrolizumab in patients with UC, keeping in mind the reduced sample size in the present study.^28^

Similar proportions of patients receiving ontamalimab or placebo reported AEs, including those considered to be related to ontamalimab, suggesting that ontamalimab has an acceptable safety and tolerability profile. Consistent with previous studies with ontamalimab,^21,24^ worsening/ongoing UC was one of the main reasons for treatment discontinuation and occurred in more patients randomised to placebo than ontamalimab. Although neoplasms were identified in the induction and maintenance studies, these were considered unrelated to ontamalimab, which was consistent with previous findings.^21,24^ A similar proportion of patients who received ontamalimab or placebo developed ADAs, and few patients developed NAbs, which suggests that this fully human monoclonal antibody was not meaningfully immunogenic in these studies.^21,24^

The safety outcomes reported in the present studies are similar to those previously observed with vedolizumab and etrolizumab in clinical trials in patients with UC.^11,26^ No confirmed cases of PML or hypersensitivity were observed with ontamalimab induction and maintenance therapy.

In terms of dose finding, ontamalimab at both 25 mg and 75 mg appears to be effective and generally tolerated as both induction and maintenance therapy for patients with UC. Although the studies were not designed to compare dosing regimens, the data suggest that induction with ontamalimab 75 mg may be more effective than ontamalimab 25 mg. For maintenance, ontamalimab 25 mg may be more effective than ontamalimab 75 mg. However, further investigations would be needed to confirm these findings for the maintenance doses. Given that patients who received ontamalimab 25 mg in the maintenance study received ontamalimab 25 mg in the induction study, it remains unknown whether 25 mg is a better choice than ontamalimab 75 mg in the maintenance phase.

Regarding study limitations, the premature discontinuation of the ontamalimab clinical trial programme in UC and CD led to a reduction in the number of patients recruited, particularly for the CD clinical trials. For CD, the low number of patients recruited means that it is not possible to characterise the efficacy and safety of ontamalimab in this patient population. In patients with UC, signals with a large level of statistical differentiation may be regarded as stable. Strengths of the studies include the use of placebo groups and two induction studies each for UC and CD, which helped to demonstrate the reproducibility of the results, and provision of the currently ongoing LTS extension study that is expected to be finalised by the end of 2023.

In conclusion, ontamalimab demonstrated a favourable benefit-risk profile as both induction and maintenance therapy when administered at doses of 25 mg and 75 mg to patients with moderate-to-severe UC. Although clinical development of ontamalimab was discontinued, the clinical trial data generated in the six studies reported suggest that ontamalimab may be a promising therapy for patients with IBD.

## Supplementary Material

jjad199_suppl_Supplementary_Tables

## Data Availability

The datasets, including the redacted study protocol, redacted statistical analysis plan, and individual participants’ data supporting the results reported in this article, will be made available within 3 months from initial request, to researchers who provide a methodologically sound proposal. The data will be provided after de-identification, in compliance with applicable privacy laws, data protection, and requirements for consent and anonymisation. Individual data and relevant materials for those patients who have consented have been transferred to and can be accessed at the Crohn’s and Colitis Foundation of America.
