# Supplementary material for: Efficacy and Safety of the Anti-mucosal Addressin Cell Adhesion Molecule-1 Antibody Ontamalimab in Patients with Moderate-to-Severe Ulcerative Colitis or Crohn’s Disease
Source: J Crohns Colitis. 2023 Dec 14;18(5):708–19. doi: 10.1093/ecco-jcc/jjad199 (PMC11140626; doi:10.1093/ecco-jcc/jjad199)
Supplement: jjad199_suppl_Supplementary_Tables [file jjad199_suppl_supplementary_tables.pdf]

Efficacy and Safety of the Anti-Mucosal Addressin Cell Adhesion Molecule-1 Antibody Ontamalimab in Patients with Moderate-to-Severe Ulcerative Colitis or Crohn's Disease

Séverine Vermeire,[a] Silvio Danese,[b] William J. Sandborn,[c] Stefan Schreiber,[d] Stephen Hanauer,[e] Geert D'Haens,[f] Peter Nagy,[g*] Manoj Thakur,[h†] Caleb Bliss,[h‡] Fabio Cataldi,[h§] Martina Goetsch,[g||] Kenneth J. Gorelick,[i] Walter Reinisch[j]

Supplementary Materials

Supplementary Methodology

Definitions of clinical response

For other secondary efficacy endpoints, remission was defined as a total Mayo score of ≤2 with no individual subscore (stool frequency [SF], rectal bleeding [RB], endoscopy and Physician Global Assessment [PGA]) exceeding 1 at week 52. Clinical response was based on total Mayo score at the week 52 visit. Clinical response [Mayo] is defined as a decrease from induction study baseline in the total Mayo score of ≥3 points and ≥30%, with an accompanying decrease in the subscore for RB of ≥1 point or an absolute RB subscore of 0 or 1. Partial Mayo remission is defined as partial Mayo score ≤2 with no individual subscore >1 over time, and does not include the endoscopy subscore.

Statistical analysis

The global family-wise type I error rate [FWER] for the statistical tests of the primary and key secondary endpoints was controlled at 0.05 [2-sided]. To control the FWER, graphical methods discussed in Bretz *et al*. [2009] were utilised to propagate α from primary to key secondary endpoints and between the two ontamalimab treatment group and placebo comparisons. α was initially split equally at the 0.025 level [2-sided] for each of the pairwise treatment comparisons for the primary endpoint and α was propagated using a hierarchical approach to each of the key secondary endpoints within a pairwise treatment comparison. For

the induction studies, power analysis was calculated based on the χ^2 test of proportions using nQuery Advisor Version 7.0 for an individual ontamalimab dose compared with placebo. Statistical results with the *p* value and significance for the primary and each key secondary endpoint were presented. Patients with missing data at the time of endpoint or with intercurrent events (rescue therapy or treatment discontinuation) before the time of the endpoint were considered failures.

For UC induction studies, randomisation of 740 subjects (2:2:1 allocation ratio: 296 subjects in the 25 mg ontamalimab treatment group, 296 subjects in the 75 mg ontamalimab treatment group, and 148 subjects in the placebo group) would yield an approximately 90% power to detect individual pairwise treatment difference in the primary efficacy endpoint (assuming 5% placebo versus 16% ontamalimab). For the UC maintenance study, power calculations are based on assuming a 0.025 (2-sided) significance level for each pairwise treatment comparison; 296 subjects previously treated with 25 mg ontamalimab in induction (1:1 allocation ratio: 148 subjects in 25 mg ontamalimab treatment group vs. 148 subjects in the placebo group) and 296 subjects treated with 75 mg ontamalimab in induction (1:1 allocation ratio: 148 subjects in the 75 mg ontamalimab treatment group vs. 148 subjects in the placebo group) were planned. These numbers would yield an approximately 98% power (85% for alpha = 0.001) to detect an individual pairwise treatment difference in the primary efficacy endpoint, remission at Week 52, of 23% (assuming 15% placebo versus 38% ontamalimab).

Glucocorticoid tapering regimen in the UC studies

Glucocorticoid tapering was mandatory starting on day 1 of the maintenance study. Given that the patients were transferred from both UC induction studies, the maximum systemic glucocorticoid dose was oral prednisone 20 mg/day or equivalent and the maximum dose of topically active glucocorticoid was oral budesonide 9 mg/day or oral beclomethasone 5 mg/day. Tapering was dependent on the dose, as follows. Daily dose >10 mg: taper by 5 mg/day each 1–2 weeks. Daily dose ≤10 mg: taper by 2.5 mg/day each 1–2 weeks. Daily dose of budesonide up to a maximum of 9 mg/day: taper by 3 mg/day every 3 weeks. If it was not

possible to taper the return to UC maintenance study baseline dose, and if there was stable disease, then apply a second taper. Daily dose of prednisone >5 mg: taper by 2.5 mg/day each week. When the daily dose of prednisone is ≤5 mg: taper by 1 mg/day each week. If unable to taper, then return to last effective dose: if >10 mg, exit study; if ≤10 mg, remain as failure for glucocorticoid-free population. To avoid confounding this study with rescue treatment, changes or additions to background therapy for UC were not permitted [except for glucocorticoid tapering and dose reductions]. Note that after tapering the glucocorticoid, any re-introduction of glucocorticoid would result in treatment failure.

Bretz F, Maurer W, Brannath W, Posch M 2009. A graphical approach to sequentially rejective multiple test procedures. *Stat Med.* 28(4): 586–604.

Supplementary Table 1. Countries, patient counts by region, and sites in the UC studies.

	**Total countries**	**Total sites**
**UC induction studies**	39	404
Patient counts by region	Eastern Europe *n* = 407<br>North America *n* = 81<br>Western Europe *n* = 71<br>Other regions *n* = 53<br>Asia *n* = 47	
**UC maintenance study**[a]	37	400

[a]No new centres were used for the maintenance studies.

UC, ulcerative colitis.

Supplementary Table 2. Inclusion and exclusion criteria in the UC studies.

**Inclusion criteria**	**Exclusion criteria**
**Ulcerative colitis induction studies**	
• ≥16 and ≤80 years of age at the time of informed consent/assent<br>• Documented diagnosis [radiologic or endoscopic with histology] of UC for ≥3 months before screening<br>• Moderate-to-severe active UC [defined as a total Mayo score of ≥6, including a centrally read endoscopic subscore ≥2, RB subscore ≥1, and stool frequency subscore ≥1 at baseline]<br>• Evidence of UC extending proximal to the rectum<br>• An inadequate response to, or lost response to, or had an intolerance to at least one conventional treatment such as mesalamine [5-aminosalicylic acid], glucocorticoids, immunosuppressants [azathioprine, 6 mercaptopurine, or methotrexate] or anti-TNF	• Indeterminate colitis, microscopic colitis, non-steroidal anti-inflammatory drug-induced colitis, ischaemic colitis, infectious colitis or clinical/histologic findings suggestive of CD<br>• Colonic dysplasia or neoplasia<br>• Colonic stricture, past medical history of colonic resection, a history of bowel surgery within 6 months before screening, or who are likely to require surgery for UC during the treatment period<br>• Refusal to receive colonoscopy during screening period if patient at risk of developing colorectal cancer<br>• Prior treatment with ontamalimab<br>• Receipt of anti-TNF treatment within 60 days before baseline, any biologic with immunomodulatory properties [other than anti-TNFs] within 90 days before baseline, or any non-biologic treatment with

	immunomodulatory properties [other than their current background UC treatment] within 30 days before baseline<br>• Prior treatment with anti-integrin/adhesion molecule treatment
**Ulcerative colitis maintenance study**	
• A decrease from the induction study baseline in the composite score of patient-reported symptoms using daily electronic diary and centrally read endoscopy of at least 2 points and at least 30%, with an accompanying decrease in the RB subscore of ≥1 point or an RB subscore of ≤1<br>OR<br>• A decrease from the induction study baseline in total Mayo score of at least 3 points and at least 30%, with an accompanying decrease in the RB subscore of at least 1 point or an absolute RB subscore of 0 or 1	• Major protocol deviation or permanent treatment discontinuation due to an adverse event in one of the induction studies<br>• Patients likely to require surgery for UC during the study period<br>• Newly diagnosed or recurrent malignancy<br>• Major illness or unstable clinical condition or any other severe acute or chronic medical condition that may increase the risk associated with study participation<br>• Known exposure to *Mycobacterium tuberculosis* since testing at screening in induction study

Anti-TNF, anti-tumour necrosis factor; NRS, numerical rating scale; RB, rectal bleeding; UC, ulcerative colitis.

Patients were excluded from the studies if they met one or more exclusion criteria.

Supplementary Table 3. Primary and secondary endpoints in the UC studies.

Endpoint		Definition
Primary	Clinical remission at week 12 [induction] or week 52 [maintenance]	A composite score of stool frequency subscore of 0 or 1 with at least a 1-point change from induction study baseline, rectal bleeding subscore of 0, and endoscopic subscore of 0 or 1 [modified, excludes friability] reported by patients using daily e-diary and centrally read endoscopy [i.e. the adapted Mayo score].
Key secondary	Endoscopic improvement	Centrally read endoscopic subscore 0 or 1.
	Symptomatic remission	Stool frequency subscore of 0 or 1 with at least a 1-point change from baseline in stool frequency subscore, and rectal bleeding subscore of 0.
	Clinical response	A decrease from induction study baseline in the composite score of patient-reported symptoms and centrally read endoscopy of ≥2 points and ≥30%, with an accompanying decrease in the subscore for rectal bleeding ≥1 point or a subscore for rectal bleeding ≤1 without rescue therapy and discontinuation; or a decrease from induction study baseline in total Mayo score of ≥3 points and ≥30%, with an accompanying decrease in the rectal bleeding subscore of at least 1 point or an absolute rectal bleeding subscore of 0 or 1.
	Mucosal healing	Centrally read endoscopic subscore 0 or 1 and a centrally read Geboes score of ≤2. Two biopsy samples were collected from the most inflamed area of the sigmoid colon at screening, at Week 12 and Week 52.
Key secondary [maintenance study]	Sustained remission	Remission at the week 52 visit among patients who were in remission at the time of baseline in the maintenance study.
	Glucocorticoid-free symptomatic remission [among patients using glucocorticoids at baseline]	Symptomatic remission at week 52 in addition to not requiring any treatment with glucocorticoids for at least 4 weeks prior to the week 52 visit.

	Glucocorticoid-free clinical remission [among patients using glucocorticoids at baseline]	Clinical remission at week 52 in addition to not requiring any treatment with glucocorticoids for at least 4 weeks prior to the week 52 visit.

UC, ulcerative colitis.

Supplementary Table 4. Change in biomarkers from baseline in patients with UC. [pharmacodynamic set]

	Induction study 1			Induction study 2			Maintenance study		
	**Placebo *N* = 74**	**Ontamalimab 25 mg *N* = 150**	**Ontamalimab 75 mg *N* = 146**	**Placebo *N* = 54**	**Ontamalimab 25 mg *N* = 111**	**Ontamalimab 75 mg *N* = 109**	**Placebo *N* = 167**	**Ontamalimab 25 mg *N* = 93**	**Ontamalimab 75 mg *N* = 98**
**Faecal calprotectin [mg/kg]**									
Baseline									
n	66	135	121	48	90	93	155	86	91
Mean [SD]	3599.37 [4258.040]	3105.46 [3441.331]	3188.27 [4064.820]	3731.29 [5103.762]	3498.90 [5350.708]	3217.33 [4020.510]	835.82 [1480.849]	1368.03 [2874.965]	824.14 [1674.688]
Week 12									
Observed value									
n	67	127	133	48	99	98	153	86	91
Mean [SD]	2172.79 [3307.686]	1933.66 [3211.360]	1560.44 [3709.552]	3908.44 [5916.298]	1791.66 [3190.469]	1275.68 [2030.308]	1686.36 [2904.412]	903.71 [1781.185]	900.59 [3373.604]
Change from baseline									
n	61	116	111	43	82	87	141	80	85
Mean [SD]	−1604.14 [4169.013]	−978.75 [4624.905]	−1630.28 [5319.024]	68.35 [7938.947]	−1598.86 [4106.382]	−1737.29 [3904.443]	682.51 [2816.635]	−474.87 [3068.558]	166.42 [3643.916]
p value	-	0.982	0.703	-	<0.001	<0.001	-	-	-
Week 52									
Observed value									
n	-	-	-	-	-	-	72	69	75

Mean [SD]	-	-	-	-	-	-	1299.33 [2109.157]	470.19 [1164.851]	482.11 [780.221]
Change from baseline									
n	-	-	-	-	-	-	70	65	71
Mean [SD]	-	-	-	-	-	-	148.38 [2019.992]	−748.58 [3200.975]	−78.84 [1066.621]
p value	-	-	-	-	-	-	-	-	-
**Serum CRP [mg/L]**									
Baseline									
n	73	149	143	54	108	108	162	91	98
Mean [SD]	10.79 [27.079]	12.15 [32.258]	6.19 [12.785]	6.57 [12.935]	10.14 [26.593]	8.56 [12.331]	3.37 [8.064]	2.34 [2.946]	4.30 [9.730]
Week 12									
Observed value									
n	68	139	140	50	101	105	160	92	97
Mean [SD]	7.43 [15.108]	5.52 [13.961]	4.33 [8.061]	9.19 [17.455]	5.14 [10.693]	5.54 [10.946]	4.92 [9.991]	2.41 [3.180]	3.48 [6.518]
Change from baseline									
n	67	138	139	50	99	104	156	90	97
Mean [SD]	−3.72 [31.332]	−3.92 [27.565]	−1.86 [13.467]	2.52 [13.983]	−2.30 [10.132]	−2.50 [12.500]	1.49 [9.551]	0.04 [2.780]	−0.82 [11.364]
p value	-	0.265	0.122	-	0.026	0.036			
Week 52									
Observed value									
n	-	-	-	-	-	-	78	73	79
Mean [SD]	-	-	-	-	-	-	4.86 [8.630]	2.84 [5.029]	2.74 [5.369]

Change from baseline									
n	-	-	-	-	-	-	77	73	79
Mean [SD]	-	-	-	-	-	-	0.63 [11.253]	0.47 [4.098]	−1.42 [11.872]
p value	-	-	-	-	-	-	-	-	-
**Serum soluble MAdCAM [pmol/L]**									
Baseline									
n	72	149	144	54	109	108	-	-	-
Mean [SD]	237.776 [138.7152]	201.703 [110.0413]	208.025 [128.5153]	238.870 [161.5773	240.349 [162.6472]	240.359 [149.6190]	-	-	-
Week 12									
Observed value									
n	66	139	141	50	102	106	-	-	-
Mean [SD]	229.089 [167.4590]	32.366 [40.6454]	13.754 [11.7090]	283.306 [184.9704]	33.010 [70.4759]	15.166 [12.3291]	-	-	-
Change from baseline									
n	64	138	140	50	100	105			
Mean [SD]	3.864 [74.7917]	−168.218 [108.2920]	−194.802 [123.1829]	39.866 [84.2060]	−207.897 [142.5458]	−223.339 [142.6712]	-	-	-
p value	-	<0.001	<0.001	-	<0.001	<0.001	-	-	-

CRP, C-reactive protein; MAdCAM, mucosal addressin cell adhesion molecule; pmol/L, picomole(s) per litre; SD, standard deviation; UC, ulcerative colitis.

Supplementary Table 5. Anti-drug antibody results for patients with UC at each visit by treatment group [safety set].

Time point	Induction study 1			Induction study 2			Maintenance study		
	**Placebo [*N* = 76] *n* [%]**	**Ontamalimab 25 mg [*N* = 151] *n* [%]**	**Ontamalimab 75 mg [*N* = 151] *n* [%]**	**Placebo [*N* = 56] *n* [%]**	**Ontamalimab 25 mg [*N* = 111] *n* [%]**	**Ontamalimab 75 mg [*N* = 112] *n* [%]**	**Placebo *N* = 170**	**Ontamalimab 25 mg *N* = 93**	**Ontamalimab 75 mg *N* = 103**
**Baseline**									
n[a]	74	149	149	56	109	111	162	93	101
ADA positive	2 [2.6]	6 [4.0]	9 [6.0]	4 [7.1]	13 [11.7]	4 [3.6]	9 [5.3]	[2 [2.2]	5 [4.9]
**Week 12**									
n[a]	68	141	139	50	98	105	164	90	94
ADA positive	2 [2.6]	7 [4.6]	3 [2.0]	5 [8.9]	8 [7.2]	4 [3.6]	17 [10.0]	6 [6.5]	8 [7.8]
**Week 52**									
n[a]	-	-	-	-	-	-	63	68	71
ADA positive							8 [4.7]	3 [3.2]	5 [4.9]
**Follow-up**									
n[a]	3	11	9	6	8	3	-	-	-
ADA positive	1 [1.3]	0 [0.0]	0 [0.0]	1 [1.8]	0 [0.0]	0 [0.0]			

[a]*n* is the number of patients with ADA positive or negative results at that time point.

ADA, anti-drug antibody; UC, ulcerative colitis.

Supplementary Information on Crohn's Disease Studies

Study design

Two similarly designed induction studies were performed in patients with CD ([NCT03559517 and NCT03566823; 278 sites in 37 countries]). A summary of patient flow in these studies is given in **Supplementary Figure 1**. Patients participating in the induction studies entered a screening period of up to 6 weeks after which eligible patients with CD could enter a 16-week treatment period. During the treatment period, patients were randomised 3:3:2 to receive either ontamalimab 25 mg, ontamalimab 75 mg or placebo via subcutaneous injections, once every 4 weeks. Patients in the CD induction studies were at least 16 years of age but younger than 80 years at the time of informed consent/assent; had a documented diagnosis of moderate-to-severe CD at least 3 months before screening; and had an inadequate response to, a lost response to, or an intolerance to at least one conventional treatment, such as mesalamine, an immunosuppressant, or an anti-tumour necrosis factor [anti-TNF] agent. Further inclusion and exclusion criteria are provided in **Supplementary Table 6**. To participate in the maintenance studies, patients had to have completed treatment in the induction studies and had to have achieved a clinical response. Patients who achieved clinical response at the end of induction could enter the maintenance study [NCT03627091; 278 sites in 34 countries with no new centres used for the maintenance studies]. Patients who did not show a clinical response at the end of the induction treatment period could participate in a long-term safety [LTS] extension study [NCT03283085; trial is ongoing]. Patients who withdrew early from the treatment period or who did not wish to enter the maintenance or LTS extension study continued into a 16-week safety follow-up period.

For patients who entered the maintenance studies [treatment for up to 52 weeks], week 16 visits from the induction studies were used as maintenance study baselines. During the treatment period, patients who had previously received ontamalimab during induction study were randomised 1:1 to receive ontamalimab [at their previous dose] or placebo. Patients who had received placebo in the induction study were randomised 2:2:1 in the maintenance study

to receive ontamalimab 25 mg, ontamalimab 75 mg or placebo [these patients did not contribute to primary efficacy analyses but contributed to safety analyses]. Patients who completed the maintenance study could enter the LTS extension study. Patients who experienced disease worsening/treatment failure (defined by protocol as sustained symptomatic worsening, confirmed by endoscopy) had to exit the study and enter the LTS to receive active treatment. Patients who discontinued early or did not enter the LTS extension study entered a 16-week safety follow-up period [reduced to 12 weeks in the LTS extension study].

Stratification in the CD induction studies was based on prior anti-TNF treatment [naive or experienced] and glucocorticoid use at baseline and Simple Endoscopic Score for CD [SES-CD] at baseline [SES-CD ≥17 or SES-CD <17]. In addition to prior anti-TNF treatment and glucocorticoid use at baseline, clinical remission by two-item patient-reported outcome [achieved or not] and enhanced endoscopic response in the induction study were stratification factors in the CD maintenance study.

Assessments and outcomes

The co-primary efficacy endpoints were clinical remission and endoscopic response at week 16 [induction studies] or week 52 [maintenance study]. Clinical remission was defined as a subscore of ≤3/11 on a two-item PRO assessing average worst daily abdominal pain in the past 7 days, as well as an average daily SF ≤2 of type 6/7, assessed by the BSFS, in the past 7 days. Endoscopic response was defined as a decrease in SES-CD of ≥25% in the induction study (at week 16) and ≥50% in the maintenance study (“enhanced” endoscopic response at week 52). Biomarker and pharmacodynamic endpoints were not analysed for the CD studies owing to the early termination of the studies and low sample size. The safety evaluation was done as per the UC studies [with the same endpoints]. Definitions for all secondary endpoints are given in **Supplementary Table 7**. Owing to the early discontinuation of the study and limited sample size in the CD studies, only descriptive statistics summaries were performed.

Definitions of clinical response

A clinical response by patients with CD was defined as a decrease of ≥30% and ≥2 points from baseline in the average daily worst abdominal pain over the seven most recent days, with the average daily SF of type 6/7 [very soft stools/liquid stools] either not worsening from baseline and/or meeting the criteria for clinical remission; or a decrease of ≥30% from baseline in the average daily SF of type 6/7 [very soft stools/liquid stools], as shown in the Bristol Stool Form Scale, over the seven most recent days, with the average daily worst abdominal pain either not worsening from baseline and/or meeting the criteria for clinical remission.

Results

Patient disposition, baseline demographics and characteristics

Overall, 63 and 40 patients were included in the induction [CD induction study 1, *n* = 29; CD induction study 2, *n* = 34] and maintenance studies, respectively. Patients were 16–72 years of age. Baseline demographics and characteristics of patients with CD are summarised in **Supplementary Table 8**.

Co-primary endpoints

In general, fewer patients with CD receiving ontamalimab (25 mg, 22.2% [*n* = 2/9] and 33.3% [*n* = 5/15]; and 75 mg, 8.3% [*n* = 1/12] and 38.5% [*n* = 5/13]) achieved clinical remission than patients receiving placebo (12.5% [*n* = 1/8] and 50.0% [*n* = 3/6]) at week 16 [**Supplementary Figure 2**]. In the two induction studies, more patients receiving ontamalimab (25 mg, 44.4% [*n* = 4/9] and 66.7% [*n* = 10/15]; 75 mg, 41.7% [*n* = 5/12] and 76.9% [*n* = 10/13]) achieved an endoscopic response at week 16 than patients receiving placebo (37.5% [*n* = 3/8] and 33.3% [*n* = 2/6]; **Supplementary Figure 3**).

At week 52 of the maintenance study, more patients who continued to receive ontamalimab (25 mg, 30.0% [*n* = 3/10] and 75 mg, 45.5% [*n* = 5/11]) achieved clinical remission than patients receiving placebo (10.5% [n = 2/19]).

At week 52 of the maintenance study, more patients who continued to receive ontamalimab 25 mg (40.0% [*n* = 4/10]) and 75 mg (54.5% [*n* = 6/11]) achieved endoscopic response than patients who received placebo (10.5% [*n* = 2/19]).

Secondary endpoints

Data for the key secondary efficacy endpoints for patients with CD are summarised in **Supplementary Table 9**.

Safety

No cases of PML were identified in the induction or maintenance studies. One potential treatment-emergent hypersensitivity event [allergic dermatitis] was reported for one patient [10.0%] receiving ontamalimab 25 mg in the maintenance study. Further data for the key safety endpoints are summarised in **Supplementary Table 10**.

Anti-drug antibodies

No more than 25.0% of patients in each group were ADA positive at each visit [**Supplementary Table 11**].

Discussion

Ontamalimab was numerically better than placebo in terms of endoscopic response. Few differences were found in rates of AEs between CD patients receiving ontamalimab or placebo, although slightly more patients who received ontamalimab 75 mg than ontamalimab 25 mg reported AEs in the maintenance study. CD patients receiving ontamalimab had more AEs considered to be associated with ontamalimab than patients who received placebo. No cases of PML and only one hypersensitivity reaction to ontamalimab were reported.

Supplementary Figures and Tables

Supplementary Figure 1.

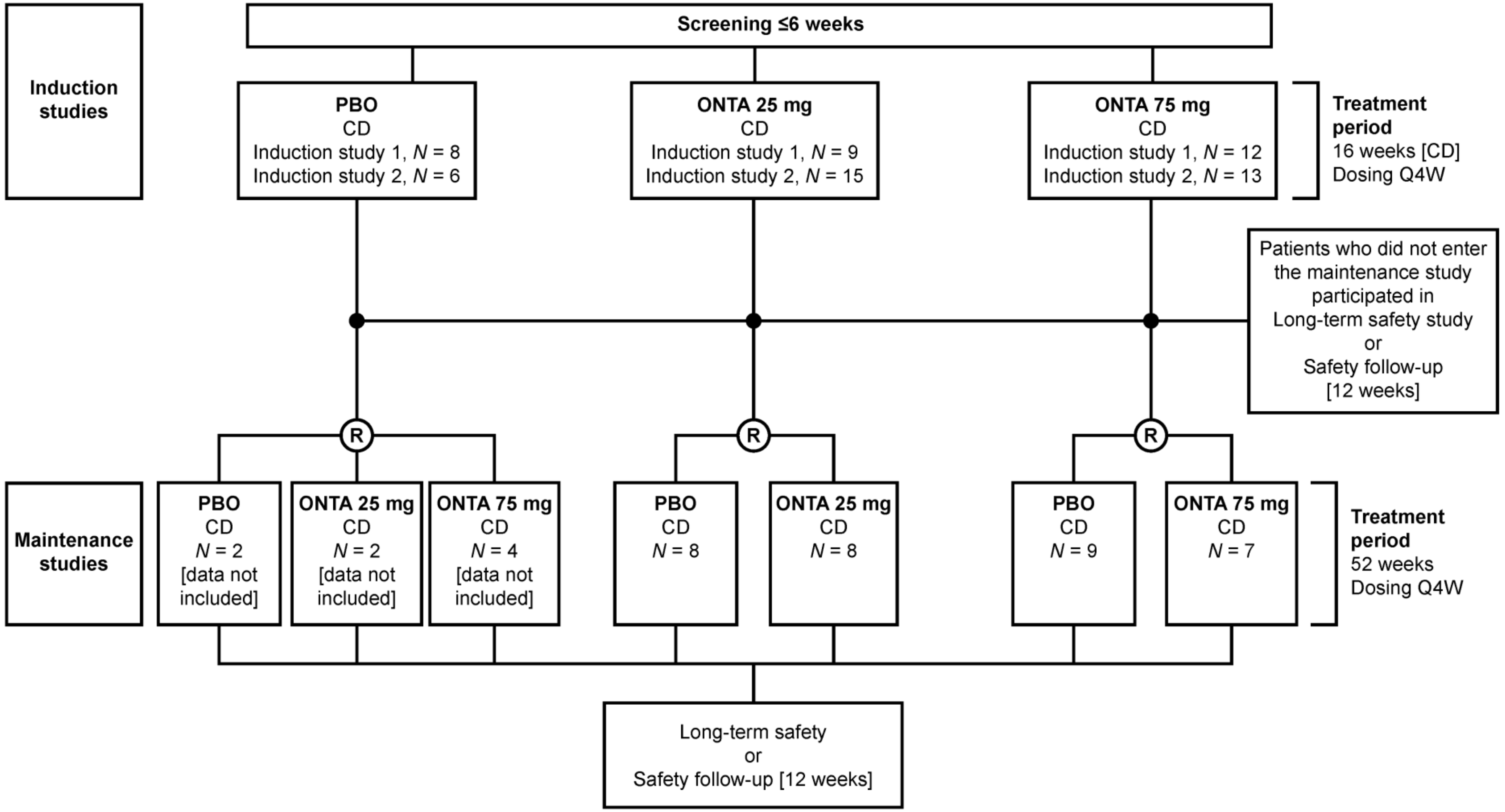

Supplementary Figure 2.

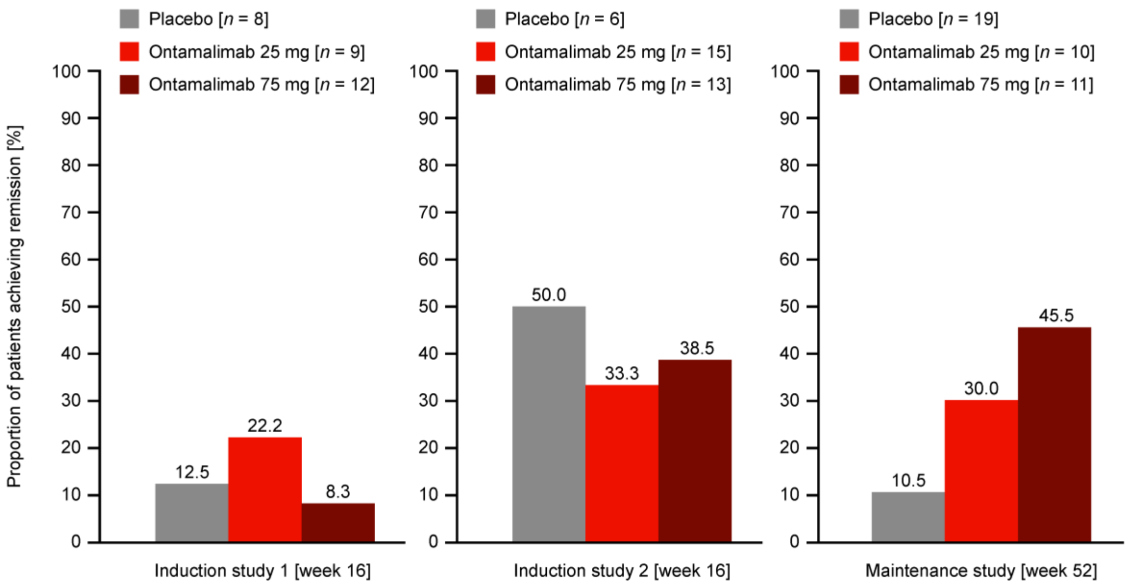

Supplementary Figure 3.

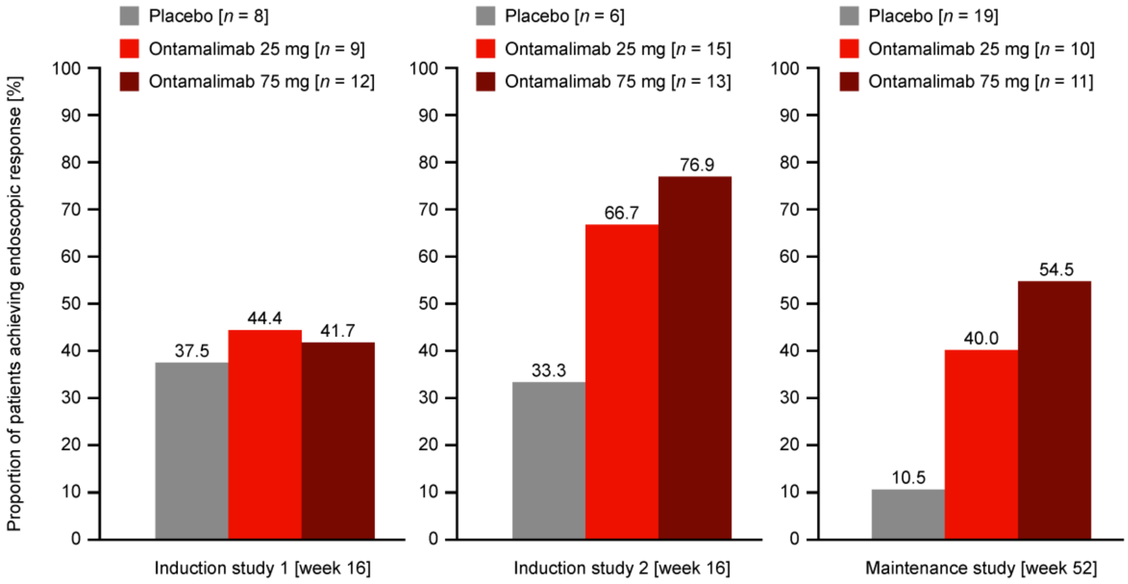

Supplementary Table 6. Inclusion and exclusion criteria in the CD studies.

**Inclusion criteria**	**Exclusion criteria**
• ≥16 and ≤80 years of age at the time of informed consent/assent<br>• Active moderate-to-severe ileal [terminal ileum], ileocolic or colonic CD at baseline as defined by both:<br>• CDAI score between 220 and 450<br>• Meeting at least one of the following subscores in the 2-item PRO<br>− Abdominal pain subscore ≥5 [average worst daily pain on the 11-point NRS] and abdominal pain subscore ≥2 [average daily pain on the 4-point abdominal pain variable of CDAI] over the 7 most recent days out of the 10 days before colonoscopy preparation<br>− Average of the daily stool frequency subscore ≥4 of type 6/7 [very soft stools/liquid stools] as shown in the BSFS over the 7 most recent days out of the 10 days before colonoscopy preparation	• Patients with indeterminate colitis, microscopic colitis, non-steroidal anti-inflammatory drug-induced colitis, ischaemic colitis, infectious colitis, or clinical/histologic findings suggestive of UC<br>• Colonic dysplasia or neoplasia<br>• Clinically significant obstructive colonic stricture, history of bowel surgery within 6 months before screening, or who are likely to require surgery for CD during the treatment period<br>• Prior treatment with ontamalimab<br>• Receipt of anti-TNF treatment within 60 days before baseline, any biologic with immunomodulatory properties [other than anti-TNFs] within 90 days before baseline, or any non-biologic treatment with immunomodulatory properties [other than their current background UC treatment] within 30 days before baseline<br>• Prior treatment with anti-integrin/adhesion molecule treatment

• Presence of ulcerations characteristic of CD [SES-CD >6 [SES-CD ≥4 for isolated ileitis]] – determined by a colonoscopy performed during screening<br>• A documented diagnosis of CD for ≥3 months before screening, as defined by both:<br>− a biopsy report in which the description of the histological findings is consistent with the CD diagnosis<br>− a report documenting disease duration based upon prior colonoscopy	
**Crohn's disease maintenance study**	
Completed the 16-week induction treatment period from the induction study<br>• Endoscopic response criteria: reduction by ≥25% in SES-CD from the induction study baseline to week 16 of the induction study<br>• At least 1 of the following 4 criteria at baseline, in addition to no worsening of endoscopic score as measured by SES-CD relative to the induction study baseline<br>− Achieving clinical remission as determined by meeting the criteria for clinical remission using the 2-item PRO, i.e., 2-item	• Major protocol deviation or permanent treatment discontinuation due to an adverse event in one of the induction studies<br>• Patients likely to require surgery for CD during the study period [except minor interventions such as seton placement for anal fistulas]<br>• Obstructive colonic stricture, or enterovesical or enterovaginal fistulae during the induction studies<br>• Newly diagnosed or recurrent malignancy

PRO subscores of average worst daily abdominal pain ≤3 [based on 11-point NRS] over the 7 most recent days* and average daily stool frequency ≤2 of type 6/7 [very soft stools/liquid stools] as shown in the BSFS over the 7 most recent days<br>− A decrease of at least 100 points in CDAI score [CDAI-100] from the induction study baseline<br>− A decrease of ≥30% and at least 2 points from the induction study baseline in the average daily worst abdominal pain over the 7 most recent days*, with the average daily stool frequency of type 6/7 [very soft stools/liquid stools] either: [i] not worsening from the induction study baseline and/or [ii] meeting the criteria for clinical remission, i.e. 2-item PRO subscore of average daily stool frequency ≤2 of type 6/7 [very soft stools/liquid stools] as shown in the BSFS over the 7 most recent days<br>− A decrease of ≥30% from the induction study baseline in the average daily stool frequency of type 6/7 [very soft stools/liquid stools] as shown in the BSFS over the 7 most recent days, with	• Major illness or unstable clinical condition or any other severe acute or chronic medical condition that may increase the risk associated with study participation<br>• Known exposure to *Mycobacterium tuberculosis* since testing at screening in induction study<br>• Any of the following abnormalities in haematology and/or serum chemistry profiles during the final induction study visit<br>− Alanine aminotransferase and aspartate aminotransferase levels ≥3.0 × ULN<br>− Total bilirubin level ≥1.5 × ULN or >2.0 × ULN if the patient has a known documented history of Gilbert's syndrome<br>− Haemoglobin level ≤80 g/L [8.0 g/dL]<br>− Platelet count ≤100 × 10^9/L [100 000 cells/mm^3] or ≥1000 × 10^9/L [1 000 000 cells/mm^3]*<br>− White blood cell count ≤3.5 × 10^9/L [3500 cells/mm^3]<br>− Absolute neutrophil count <2 ×10^9/L [<2000 cells/mm^3]

the average daily worst abdominal pain either not worsening from the induction baseline and/or meeting the criteria for clinical remission [2-item PRO subscore of average worst daily abdominal pain ≤3 based on 11-point NRS] over the 7 most recent days	− Serum creatinine level >1.5 × ULN or estimated glomerular filtration rate <30 mL/min/1.73 m^2 based on the abbreviated Modification of Diet in Renal Disease Study Equation<br>− *Note: if platelet count is <150 000 cells/mm^3, a further evaluation should be performed to rule out cirrhosis, unless another aetiology has already been identified

Anti-TNF, anti-tumour necrosis factor; BSFS, Bristol Stool Form Scale; CD, Crohn's disease; CDAI, Crohn's disease Activity Index; NRS, numerical rating scale; PRO, patient-reported outcome; SES-CD, Simple Endoscopic Score for Crohn's disease; ULN, upper limit of normal.

Patients were excluded from the studies if they met one or more exclusion criteria.

Supplementary Table 7. Primary and secondary endpoints in the CD studies.

Endpoint		Definition
Co-primary	Clinical remission at weeks 16/52	A two-item PRO subscore of average daily stool frequency ≤2 of type 6/7 [very soft stools/liquid stools] as shown in the BSFS over the 7 most recent days and a subscore of ≤3/11 on a two-item PRO assessing average worst daily abdominal pain.
	Endoscopic response at week 16 or enhanced endoscopic response at week 52	A decrease in SES-CD of ≥25% or ≥50% from baseline at week 16 and week 52 respectively.
Secondary	Symptomatic remission based on CDAI score at week 16/52	A CDAI score of <150.
	Enhanced endoscopic response at week 16 and sustained enhanced endoscopic response at week 52	Enhanced endoscopic response: a decrease in SES-CD of ≥50% from baseline.
	Symptomatic remission based on a two-item PRO with 4-point scale for abdominal pain at week 16/52	Symptomatic remission based on a two-item PRO with 4-point scale for abdominal pain: Average Daily Abdominal Pain ≤1 (Based on the 4-point Scale) and Average Daily Stool Frequency ≤3 of Type 6/7.
	Clinical response based on a two-item PRO with two criteria at week 16/52	Clinical response meeting at least one of the following two criteria:<br>1. A decrease of ≥30% and at least 2 points from baseline in the average daily worst abdominal pain over the 7 most recent days*, with the average daily stool frequency of type 6/7 (very soft stools/liquid stools) either:<br>(a) Not worsening from baseline<br>and/or<br>(b) Meeting the criteria for clinical remission, i.e., 2 item PRO subscore of average daily stool frequency ≤2 of type 6/7 (very soft stools/liquid stools) as shown in the BSFS over the 7 most recent days

		2. A decrease of ≥30% from baseline in the average daily stool frequency of type 6/7 (very soft stools/liquid stools) as shown in the BSFS over the 7 most recent days, with the average daily worst abdominal pain either:<br>(a) Not worsening from baseline<br>and/or<br>(b) Meeting the criteria for clinical remission, i.e. 2 item PRO subscore of average worst daily abdominal pain ≤3 (based on 11-point NRS) over the 7 most recent days.
	Clinical remission by 2-item PRO and enhanced endoscopic response	2-item PRO subscores of average worst daily abdominal pain ≤3 (based on 11-point NRS) over the 7 most recent days and average daily stool frequency ≤2 of type 6/7 (very soft stools/liquid stools) as shown in the BSFS over the 7 most recent days together with a decrease in SES-CD of at least 50% from induction study baseline
	Complete endoscopic healing at week 16/52	SES-CD of 0–2.
	Glucocorticoid-free clinical remission at the Week 52 visit, among subjects using glucocorticoids at induction study baseline	clinical remission by 2-item PRO (as defined for the coprimary endpoint) in addition to not requiring any treatment with glucocorticoids for at least 12 weeks prior to the Week 52 visit
	Sustained clinical remission	in clinical remission at Week 52 visit, among subjects who were in clinical remission by 2-item PRO (as defined for the co-primary endpoint) at baseline of the maintenance study

BSFS, Bristol Stool Form Scale; CD, Crohn’s disease; CDAI, Crohn’s disease Activity Index; PRO, patient-reported outcome; SES-CD, Simple Endoscopic Score for Crohn’s disease.

Supplementary Table 8. Baseline demographics and characteristics for patients with Crohn's disease [full analysis set].

	Induction study 1			Induction study 2			Maintenance study		
	**Placebo** ***N*** **= 8**	**Ontamalimab 25 mg** ***N*** **= 9**	**Ontamalimab 75 mg** ***N*** **= 12**	**Placebo** ***N*** **= 6**	**Ontamalimab 25 mg** ***N*** **= 15**	**Ontamalimab 75 mg** ***N*** **= 13**	**Placebo** ***N*** **= 19**	**Ontamalimab 25 mg** ***N*** **= 10**	**Ontamalimab 75 mg** ***N*** **= 11**
**Age [years]**[a]									
Mean [SD]	40.0 [13.8]	40.1 [15.6]	38.1 [17.7]	42.4 [13.6]	37.5 [10.2]	39.1 [12.7]	37.7 [14.26]	38.4 [7.18]	45.8 [14.85]
Min, max	19, 58	19, 72	16, 69	26, 62	18, 55	19, 57	17, 64	28, 53	21, 69
**Sex,** ***n*** **[%]**									
Male	3 [37.5]	5 [55.6]	7 [58.3]	4 [66.7]	7 [46.7]	8 [61.5]	12 [63.2]	4 [40.0]	6 [54.5]
**Race,** ***n*** **[%]**									
Asian	0 [0.0]	0 [0.0]	0 [0.0]	0 [0.0]	2 [13.3]	0 [0.0]	1 [5.3]	0 [0.0]	0 [0.0]
Black or African American	0 [0.0]	0 [0.0]	0 [0.0]	1 [16.7]	0 [0.0]	1 [7.7]	1 [5.3]	0 [0.0]	0 [0.0]
White	8 [100.0]	9 [100.0]	12 [100.0]	3 [50.0]	12 [80.0]	11 [84.6]	17 [89.5]	8 [80.0]	10 [90.9]
Other	0 [0.0]	0 [0.0]	0 [0.0]	2 [33.3]	1 [6.7]	1 [7.7]	0 [0.0]	2 [20.0]	1 [9.1]
**Disease duration, years**[b]									
Mean [SD]	7.2 [7.0]	4.0 [4.2]	9.9 [11.4]	3.3 [3.0]	6.1 [7.5]	8.9 [7.6]	8.8 [10.0]	4.9 [5.2]	9.7 [10.3]
**Baseline faecal calprotectin** [mg/kg]									
n	6	7	11	5	14	12	-	-	-
Mean [SD]	2378.0 [1768.1]	3932.6 [3699.8]	3705.3 [3231.3]	2364.1 [2369.0]	3692.4 [4822.1]	2774.7 [3217.0]	-	-	-
**Status of anti-TNF experienced**[d], *n* [%]									

Experienced	4 [50.0]	3 [33.3]	7 [58.3]	2 [33.3]	7 [46.7]	8 [61.5]	9 [47.4]	2 [20.0]	5 [45.5]
Naive	4 [50.0]	6 [66.7]	5 [41.7]	4 [66.7]	8 [53.3]	5 [38.5]	10 [52.6]	8 [80.0]	6 [54.5]
**Anti-TNF failure,**[e] *n* [%]									
	4 [50.0]	2 [22.2]	3 [25.0]	1 [16.7]	6 [40.0]	7 [53.8]	7 [36.8]	2 [20.0]	4 [36.4]
**GC use at baseline**,[d] *n* [%]									
	2 [25.0]	4 [44.4]	5 [41.7]	1 [16.7]	6 [40.0]	9 [69.2]	9 [47.4]	2 [20.0]	5 [45.5]
**Maximum prior treatment experience**[f], *n* [%]									
5-ASA experienced	0 [0.0]	0 [0.0]	0 [0.0]	0 [0.0]	0 [0.0]	0 [0.0]	0 [0.0]	0 [0.0]	0 [0.0]
GC experienced	2 25.0]	1 [11.1]	1 [8.3]	2 [33.3]	3 [20.0]	3 [23.1]	4 [21.1]	1 [10.0]	5 [45.5]
Topical GC experienced	0 [0.0]	0 [0.0]	0 [0.0]	0 [0.0]	0 [0.0]	2 [15.4]	1 [5.3]	0 [0.0]	1 [9.1]
Systemic GC experienced	2 [25.0]	1 [11.1]	1 [8.3]	2 [33.3]	3 [20.0]	1 [7.7]	3 [15.8]	1 [10.0]	4 [36.4]
Immunosuppressant experienced or biologic failure	3 [37.5]	5 [55.6]	10 [83.3]	4 [66.7]	8 [53.3]	5 [38.5]	10 [52.6]	7 [70.0]	4 [36.4]
Immunosuppressant experienced and biologic failure	3 [37.5]	3 [33.3]	1 [8.3]	0 [0.0]	4 [26.7]	5 [38.5]	5 [26.3]	2 [20.0]	2 [18.2]

The baseline values were the last value collected before the first dose of study treatment.

[a]One patient who was 17.84 years of age was enrolled in the ontamalimab 25 mg treatment group. The patient's age was rounded up to 18 years for the purpose of establishing a minimum value.

[b]Disease duration was the number of years from the date of UC diagnosis to the date of informed consent.

[d]Status of prior anti-TNF therapy and GC use at baseline were based on actual stratification levels.

[e]Anti-TNF failure included intolerance.

[f]Patients were counted once at the maximum prior treatment experienced with the categories representing increasing prior treatment experienced.

5-ASA, aminosalicylate; anti-TNF, anti-tumour necrosis factor; CD, Crohn’s disease; GC, glucocorticoid; SD, standard deviation; UC, ulcerative colitis.

Supplementary Table 9. Key secondary endpoints for patients with Crohn's disease [full analysis set].

	**Induction study 1**			**Induction study 2**			**Maintenance study**		
	**Placebo *N* = 8**	**Ontamalimab 25 mg *N* = 9**	**Ontamalimab 75 mg *N* = 12**	**Placebo *N* = 6**	**Ontamalimab 25 mg *N* = 15**	**Ontamalimab 75 mg *N* = 13**	**Placebo *N* = 19**	**Ontamalimab 25 mg *N* = 10**	**Ontamalimab 75 mg *N* = 11**
**Symptomatic remission[a] based on CDAI at week 16 [induction studies] and week 52 [maintenance study]**									
n [%]	3 [37.5]	4 [44.4]	3 [25.0]	4 [66.7]	8 [53.3]	6 [46.2]	8 [42.1]	4 [40.0]	7 [63.6]
Difference in proportion		0.07	−0.13		−0.13	−0.21		−0.02	0.22
95% CI	-	−0.34, 0.44	−0.48, 0.24	-	−0.46, 0.29	−0.53, 0.24	-	−0.34, 0.32	−0.14, 0.50
**Enhanced endoscopic response[b] at week 16 [induction studies] and sustained enhanced endoscopic response at week 52 [maintenance study]**									
n [%]	1 [12.5]	2 [22.2]	0 [0.0]	2 [33.3]	5 [33.3]	3 [23.1]	1 [5.3]	2 [20.0]	3 [27.3]
Difference in proportion		0.10	−0.13		0.00	−0.10		0.15	0.22
95% CI	-	−0.28, 0.44	−0.47, 0.14	-	−0.41, 0.34	−0.50, 0.26	-	−0.09, 0.46	−0.04, 0.52
**Symptomatic remission as measured by two-item PRO with 4-point scale for abdominal pain at week 16 [induction studies] and week 52 [maintenance study]**									
n [%]	1 [12.5]	3 [33.3]	1 [8.3]	3 [50.0]	6 [40.0]	6 [46.2]	2 [10.5]	3 [30.0]	7 [63.6]
Difference in proportion		0.21	−0.04		−010	−0.04		0.19	0.53
95% CI	-	−0.20, 0.54	−0.39, 0.25	-	−0.47, 0.30	−0.43, 0.36	-	−0.09, 0.51	0.18, 0.76
**Clinical response as measured by two-item PRO with two criteria at week 16 [induction studies] and week 52 [maintenance study]**									
n [%]	4 [50.0]	8 [88.9]	6 [50.0]	5 [83.3]	11 [73.3]	8 [61.5]	2 [10.5]	1 [10.0]	2 [18.2]
Difference in proportion		0.39	0.00		−0.10	−0.22		−0.01	0.08
95% CI	-	−0.04, 0.69	−0.38, 0.38	-	−0.39, 0.33	−0.51, 0.23	-	−0.23, 0.31	0.17, 0.38
**Symptomatic remission with endoscopic response at week 16 [induction studies] and week 52 [maintenance study]**									
n [%]	1 [12.5]	1 [11.1]	1 [8.3]	1 [16.7]	4 [26.7]	5 [38.5]	0 [0.0]	3 [30.0]	4 [36.4]

Difference in proportion		−0.01	−0.04		0.10	0.22		0.30	0.36
95% CI	-	−0.37, 0.33	−0.39, 0.25	-	−0.33, 0.39	−0.23, 0.51	-	0.04, 0.60	0.09, 0.65
**Complete endoscopic healing at week 16 [induction studies] and week 52 [maintenance study]**									
n [%]	0 [0.0]	2 [22.2]	0 [0.0]	1 [16.7]	3 [20.0]	2 [15.4]	0 [0.0]	3 [30.0]	3 [27.3]
Difference in proportion		0.22	-		0.03	−0.01		0.30	0.27
95% CI	-	−0.14, 0.55	-	-	−0.38, 0.32	−0.42, 0.29	-	0.04, 0.60	0.03, 0.57

[a]Symptomatic remission was defined as a CDAI score of <150.

[b]An enhanced endoscopic response was defined as a decrease in SES-CD of at least 50% from baseline.

CDAI, Crohn’s disease Activity Index; CI, confidence interval; PRO, patient-reported outcome; SES-CD, Simple Endoscopic Score for Crohn’s disease.

Supplementary Table 10. Adverse events reported for patients with Crohn's disease [safety set].

	Induction study [SHP647-305]			Induction study [SHP647-306]			Maintenance study		
	**Placebo** **_N_ = 8**	**Ontamalimab 25 mg _N_ = 9**	**Ontamalimab 75 mg _N_ = 12**	**Placebo** **_N_ = 6**	**Ontamalimab 25 mg _N_ = 15**	**Ontamalimab 75 mg _N_ = 13**	**Placebo** **_N_ = 19**	**Ontamalimab 25 mg _N_ = 10**	**Ontamalimab 75 mg _N_ = 11**
Any TEAE, *n* [%]	5 [62.5]	4 [44.4]	8 [66.7]	6 [100.0]	7 [46.7]	6 [46.2]	9 [47.4]	5 [50.0]	8 [72.7]
Serious TEAE, *n* [%]	1 [12.5]	0 [0.0]	3 [25.0]	0 [0.0]	2 [13.3]	2 [15.4]	1 [5.3]	0 [0.0]	2 [18.2]
Related to ontamalimab	3 [37.5]	1 [1.11]	2 [16.7]	2 [33.3]	2 [13.3]	1 [7.7]	1 [5.3]	1 [10.0]	2 [18.2]
Leading to study discontinuation	0 [0.0]	0 [0.0]	0 [0.0]	1 [16.7]	1 [6.7]	2 [15.4]	0 [0.0]	0 [0.0]	2 [18.2]
Leading to study medication discontinuation	0 [0.0]	0 [0.0]	0 [0.0]	1 [16.7]	1 [6.7]	2 [15.4]	0 [0.0]	0 [0.0]	1 [9.1]
Leading to death	0 [0.0]	0 [0.0]	0 [0.0]	0 [0.0]	0 [0.0]	0 [0.0]	0 [0.0]	0 [0.0]	0 [0.0]

TEAE, treatment-emergent adverse event.

Supplementary Table 11. Anti-drug antibody results at each visit by treatment group for patients with Crohn's disease [safety set].

Time point	Induction study [SHP647-305]			Induction study [SHP647-306]			Maintenance study		
	**Placebo [*N* = 8] *n* [%]**	**Ontamalimab 25 mg [*N* = 9] *n* [%]**	**Ontamalimab 75 mg [*N* = 12] *n* [%]**	**Placebo [*N* = 6] *n* [%]**	**Ontamalimab 25 mg [*N* = 15] *n* [%]**	**Ontamalimab 75 mg [*N* = 13] *n* [%]**	**Placebo [*N* = 19] *n* [%]**	**Ontamalimab 25 mg [*N* = 10] *n* [%]**	**Ontamalimab 75 mg [*N* = 11] *n* [%]**
**Baseline**									
n[a]	8	8	12	6	15	12	19	10	11
ADA positive	1 [12.5]	1 [11.1]	1 [8,.3]	0	2 [13.3]	3 [23.1]	2 [10.5]	0	1 [9.1]
**Week 12**									
n[a]	8	9	11	5	12	11	18	10	10
ADA positive	2 [25.0]	1 [11.1]	1 [8.3]	0	2 [13.3]	0	2 [10.5]	0	1 [9.1]
**Follow-up**									
n[a]	2	0	1	2	0	1	-	-	-
ADA positive	1 [12.5]	0	0	1 [16.7]	0	0			

[a]*n* is the number of patients with ADA positive or negative results at that time point.

ADA, anti-drug antibody.
